# A New Organization

**Published:** 1889-06

**Authors:** 


### A New Organization—A District Society.

The Seventh, or Southwestern District of Ohio for Dental Association Work, held a meeting for organization in Dayton on Tuesday, May 21st. The object was simply to organize and arrange for future meetings, etc. It was not expected that a large number would be present at this first meeting; there were fifteen present.

A draft of a constitution and by-laws was presented and after some amendment and changes was adopted. These were signed by all those present. After this an election of officers was held which resulted in the selection of Dr. E. G. Betty, of Cincinnati, President; Dr. L. E. Custer, of Dayton, Vice-President; Dr. Chas. Welch, of Wilmington, Secretary; and Dr. Chas. I. Keely, of Hamelton, Treasurer.

Executive Committee, J. Taft, Chas. Welch, L. C. Adams.

Some discussion was had in regard to the plan of work for the future and much interest was manifested for the success of the young society. The next meeting will be held on the third Tuesday of September next; the place is not yet decided, but will be soon. It is very desirable that the entire profession in the district unite in this work. Let every one come up to the next meeting prepared to do something for others as well as to have others do something for him. It is to be hoped that each of these district societies will be able to come up to the next meeting of the State society with reports that will show what Ohio can do; there will be some interest to see what district will bring up the best report.

The Seventh District consists of the following counties, viz: Adams, Brown, Clermont, Hamelton, Butler, Warren, Clinton, Highland, Fayette, Green, Montgomery and Preble. These counties contain enough good dentists to form a society of from fifty to seventy-five members, and it will be a disappointment if such a membership is not reached. We have received information that several of the other districts will organize within the next month. And it is desirable that all organize as early as possible.

